# Do hospitals need to establish multiple hospital districts? A hospital-based perspective on the benefits of scale

**DOI:** 10.3389/fpubh.2023.1019331

**Published:** 2023-03-24

**Authors:** Yuan Zheng, Zhang Yuqing, Zhang Mengping, Li Jun

**Affiliations:** ^1^School of Public Health, Capital Medical University, Beijing, China; ^2^Capital Health Management and Policy Research Base, Beijing, China; ^3^Beijing Hospital of Traditional Chinese Medicine, Beijing, China; ^4^Beijing Hospitals Authority, Beijing, China

**Keywords:** hospital bed capacity, hospital planning, model, hospital administration, hospital management

## Abstract

**Background:**

During the fight against COVID-19, China’s public hospitals played the main role in taking on the most urgent, dangerous and arduous medical treatment and work. Therefore, in order to promote the high-quality development of hospitals, it is necessary to support some potential public hospitals to build and develop a “One Hospital with Multiple Campuses System” (OHMC) based on controlling the size of single hospitals, and to quickly convert their functions in the event of a severe epidemic.

**Methods:**

The Cobb–Douglas production function and log-transformed production function were used to measure the appropriate hospital size for 22 public hospitals in a region of China.

**Results:**

The eight OHMC hospitals that planned to be build are basically qualified to handle the conditions and potential of multi-districts from the perspective of economy of scale. The OHMC hospitals in operation appear to have weakened incremental scale rewards, because they are in the process of development, but they are still higher than the overall level of single-campus hospitals.

**Conclusion:**

The expansion of hospital scale may bring the advantages of group development, but it may also bring about problems including rising hospital cost, increasing management and operation cost, inefficient allocation of medical resources and unbalanced development.

## Background

The One Hospital with Multiple Campuses (OHMC) system is a hospital development strategy selecting one main hospital to lead multiple branches (1, 2). This model is based on the establishment of a “core output” general hospital that provides guidance and support to the branches in terms of management, medical care and funding. In addition, these hospitals benefit from the services of the same legal person and unified financial management, simultaneously developing different areas. In late 2016, *China’s 13th Five-Year Plan for Deepening the Reform of the Medical and Health System* issued (3) by the State Council of the PRC promoted the hierarchical medical system, which graded medical institutions at different levels based on the priority of diseases and the ability to provide high-level treatment for different diseases. Therefore, hospitals are incentivized to progress from general practice to specialization, which requires reasonable control of the number and scale of public general hospitals.

The OHMC system management model was first established by the administrators of the Willis-Knighton Medical Center located in Shreveport, Louisa, in 1983 (4) due to the growing population of southern Shreveport but inadequate health facilities. Today the Williston Health System encompasses five branches in the United States. In this model, the main hospital is responsible for setting policies that the branch hospital adopts. This approach not only ensures consistency in management but also enables patients to receive similar medical services at different branches. Kosair Children’s Hospital in Louisville, Kentucky, is the only full-service children’s hospital in the region. Nichols KM et al. performed a comparative analysis of the number of patients, hospital revenue, and types of patients at Kosair Children’s Hospital and Medical Center from 2010 to 2013 (5). They found that the establishment of a branch hospital could accommodate patients from the surrounding area who previously did not have access to children’s medical care. The development of the branch hospital did not cause a significant decline in the number of patients at the main hospital, thus showing a positive impact on overall hospital development.

In February 2021, the 18th meeting of the Central Committee for Comprehensively Deepening Reform considered and adopted *the Opinions on Promoting the High-Quality Development of Public Hospitals (2021)* (6). In the process of fighting the New Crown Pneumonia Pandemic, public hospitals had taken on the most urgent, dangerous and difficult medical treatment and played a key role. In order to control the size of single hospitals, some capable public hospitals should be supported by the state to properly build and develop branches so that they can quickly convert their functions in the event of another major pandemic.

China is a geographically large country, but most large general hospitals are established in urban centers. The development of multi-district hospitals can not only relieve the pressure on the central city hospitals but also meet the local healthcare needs of the general public. This is especially important in the post-pandemic era (when the pandemic is short-lived, but the impact of the pandemic is long-lasting). Exploring a single multi-district model for public hospitals could provide solutions to healthcare access and allow for the rapid conversion of hospital functions.

A study investigated whether U.S. state hospitals had sufficient beds to respond in the event of a large pandemic outbreak (7) and revealed high variability across the U.S. healthcare system. Some states had an average number of beds close to those of developing countries, which was low compared to some developed countries. Fortunately, at the time of the current COVID-19 pandemic, most of the cases were concentrated in a few states with relatively high bed counts, which somewhat attenuated the severity of the pandemic. Countries such as Germany and Austria, which have three times (8) the number of hospital beds than the United States, could have responded better to the pandemic. This evidence supports the need for OHMC in China.

According to the *Guiding Principles for Medical Institution Setting Planning (2021–2025)* (9) issued by the National Health Commission of the People’s Republic of China, hospitals should comprehensively consider the region’s economic, social, and medical resources layout along with the public’s health needs to coordinate the planning of medical resources and layout. In principle, public hospitals are encouraged to build branch hospitals to maintain control over the size of a single unit. Hospital size is generally assessed by the number of beds (10). In accordance with the *General Hospital Construction Standards (2021)* (11) announced in China, the number of hospital beds should consider the regional health planning, medical institutions set up planning, the population served, the morbidity rate and the regional economic level.

Regional health planning (12) refers to the comprehensive planning of health development and resource allocation in the region. In contrast, medical institutions planning (9) refers to the forecasting and planning of the annual medical services demand to determine the level, category, number, scale and distribution of medical institutions needed in a region. This planning is based on the requirements of graded treatment, the economic status of the region, accessibility of medical services, and the potential of medical institutions to transform into service demand. The population served (13) refers to the working population of the relevant sectors serving the city in the management area; the morbidity rate is defined as the frequency of new disease cases in a certain population within a certain period of time. The regional economic level (14) refers to the scale, speed and level of economic development achieved in the region, which is often expressed in terms of Gross Domestic Product (GDP).

Ravaghi et al. (15) argue that hospital size is usually determined by policymakers and managers at different levels of the health system. Two things are generally considered when planning the number of hospital beds: the number of beds needed for different time horizons (short, medium or long term) and the level of bed capacity planning (macro, micro and hospital operational levels). Forecasting the size of hospitals in a given area relates to the long-term needs of that area, while specific planning occurs at the hospital level. The optimal number of beds per hospital unit is determined by hospital operations (16). Changes in hospital beds are a highly sensitive political issue involving negotiations between various stakeholder parties, so economic indicators are rarely used to inform decisions (17, 18).

Some other Chinese scholars have explored the relevant factors that should be considered for hospital size, but no empirical research has been conducted on the quantitative relationship between factors and size, (19, 20) let alone explicitly proposing a specific size quantity for the OHMC. For example, Ma (21) reported that hospitals should consider the total amount of overall local health resources input and the structure of health resources when undertaking scale expansion. Yi et al. (22) and other scholars suggested that the hospital scale size should take into consideration the functional positioning and nature of this hospital; Li et al. (19) studied the hospital scale in Guangdong Province, China, and found that an excessive hospital scale might lead to increased social costs, thus increasing the cost of patient care. Furthermore, Xian-Wen et al. (23) argue that the measurement of hospital size also needs to consider the hospital’s fixed assets, internal management efficiency, and so on.

In addition, due to the public interest nature of China’s public hospitals, the government and researchers usually plan the size of hospitals from the perspective of patient demand or top-level design by government authorities based on the overall development needs of the city. In reality, however, the construction and management of the OHMC are completely different from the establishment of a new single hospital, as the OHMC also needs to consider the specific situation of the existing hospital and branches, such as whether the hospital has the capacity to build a new hospital in its current operation. Therefore, the need to establish a branch and the size of the new branch should not rely solely on theoretical calculations but also needs to be self-regulated according to the actual hospital development. This paper explores whether hospitals have the capacity to develop a multi-campus from the perspective of hospitals, taking into account the number of beds.

## Methods

### Sources of data

Data were obtained from 22 municipal public hospitals in a city in China from 2015 to 2020, including 10 general hospitals and 12 specialty hospitals: one Chinese medicine hospital, one oncology hospital, three infectious disease hospitals, two mental health specialty hospitals, two pediatric hospitals, one obstetrics and gynecology hospital, one geriatric hospital, and one dental hospital, with 110 observations. Additional data were obtained from the city’s Public Health Information Center. The intent of these 22 hospitals to develop multiple hospital districts was classified as those planning to conduct OHMC (A), those already conducting OHMC (B), and those not planning to develop OHMC (C).

### Modeling

The scale economies of hospitals refers to the phenomenon of increasing payoffs, or economic increases, as hospitals increase in size (24). The economic increase mainly manifests as a decrease in medical service output cost and an increase in the market share and value of the hospital brand. Economies of scale incentivize hospitals to expand, with a larger scale being more favorable (19). However, a large scale increases the complexity of the hospital’s internal organization. The increased cost and time to coordinate activities increases, and the ability to respond quickly to external changes decreases, resulting in diminishing returns to the hospital scale, which means that scale is not economical. Therefore, there is an appropriate size or optimal size. In economics, the scale corresponding to the economies of scale is termed the optimal scale or economic scale (25).

In 1991, Lin Zihua, Hao Mo and others (26) used the Cobb–Douglas production function to study the economies of scale of some township health hospitals and found that an appropriate increase in beds could expand the economic scale benefits. In 2004, Yan study (20) of seven general hospitals in a region found that an increase in hospital beds did not mean an increase in hospital performance. Hospital performance showed a parabolic trend, peaking at 1100 beds, indicating that either too large or too small hospital bed size is inappropriate and that there is a correlation between hospital bed size and hospital performance.

The provision of healthcare services is complex due to its strong professionalism, technicality and information asymmetry. At the same time, the health sector also involves a high number of inputs and outputs. Therefore, in recent years, more advanced econometric and mathematical methods have been introduced internationally to analyze the efficiency of organizations providing health services.

#### Production function

The production function (15) represents the relationship between the number of inputs of a factor of production and the maximum output it can produce under certain technical conditions. The two most widely used models for estimating hospital production function are the Cobb–Douglas production function Y = AL*^α^*K*^β^*, and the log-transformed production function lnY = lnA + αlnL+ βlnK, where output is denoted by Y, and A represents the total output rate coefficient (size), L represents labor factor inputs, capital factor inputs are denoted by K, the share of labor income in total output is represented by α, and the share of capital income in total output is represented by β.

#### Cost function

The cost function (27) is the minimum cost of producing a given level of output at some fixed factor price. According to the theory of producer equilibrium, the cost is divided into the total cost (TC), the average cost (AC) and the marginal cost (MC). The total cost function is TC = TFC + TVC, where TFC means the total fixed cost and TVC means the total variable cost. The average cost equation is AC = TC/Q, and the marginal cost equation is MC = ∆TC/∆Q, where Q represents quality. The type of economies of scale can be measured according to the cost-output elasticity: local economies of scale when the cost-output elasticity is less than 1; local constant scale when it is equal to 1 payoff; local diseconomies of scale when it is less than 1.

#### Data envelopment analysis

Since Farrell (15) proposed the frontier view to evaluate institutional efficiency from a relative perspective, data envelopment analysis (28) (DEA) has been introduced into health econometrics since the mid-1980s. Since then, this field has matured, and advanced methods to evaluate the technical efficiency of health institutions are now available. The resource management and service output of hospitals can be analyzed, providing a benchmark for health services research.

#### Stochastic frontier analysis

Stochastic frontier analysis (SFA) is a new method of hospital evaluation developed after data envelopment analysis (24, 29). SFA was first proposed by Faree in 1977 and then gradually refined by Aigner et al. (30) It uses a set of combined error models to measure the distance between actual production costs and frontier costs, i.e., inefficiency losses. However, the parameters estimated by the fixed effects model of SFA may be biased when assessing a short time period, but the model is still considered to be the optimal method for measuring the efficiency of multi-input–output systems (31).

China has been exploring changing hospital management models in recent years in order to promote high-quality hospital development. Compared with the other 3 methods, the production function requires fewer measurement metrics, is easier to harmonize, and has lower requirements for data quality. Therefore, the production function model was used to measure the appropriate size of hospitals, taking into account the availability and consistency of information and the actual situation of the investigated hospitals.

The two most commonly used models for estimating hospital production functions are the Cobb–Douglas production function and the log-transformed production function. The basic model of the production function is $Y=A\cdot L^{\alpha }\cdot K^{\beta }$ (15):

(1) When α + β = 1, which indicates the payoff of scale, the input quantity increases or decreases by X times, and the output quantity also increases or decreases by X times accordingly. In other words, the output effect of expanding the size of a hospital by one time is equal to that of building two hospitals of the same size.

(2) When α + β > 1, which represents incremental scale payoff, the input increases by a factor of 1 and the output increases by more than a factor of 1. In this case, increasing the size of a hospital by a factor of 1 is better than building 2 hospitals of the same size.

(3) When α + β < 1, which indicates decreasing scale payoff, the input increases by 1 time and the output increases by less than 1 time. In this case, expanding the size of one hospital by 1 time is smaller than the output of building another hospital of the same size.

α and β denote the elasticity coefficients of labor and capital, respectively. α and β represent the ratio of the intensity of the effect of labor and capital inputs on the output quantity corresponding to a given output quantity Y. The payoff of scale refers to the change in the quantity of output caused by the change in the scale of production (i.e., the change in the quantity and combination of input factors) under certain technical conditions.

Similar to the medical field, there are some qualitative factors affecting the development of China’s semiconductor industry that are difficult to quantify, including fiscal policy, fixed asset investment, R&D investment, labor input, and geographic environment. Therefore, we refer to this semiconductor industry size forecast model, (32) extending the Cobb–Douglas production function, to build a new model: $Y_{t}=A\cdot K_{t}^{\alpha }\cdot H_{t}^{\beta }\cdot L_{t}^{\gamma }$ , where α and β denote the output elasticity of capital, and γ denotes the output elasticity of labor. The natural logarithm of both sides of this production function model and the natural logarithm of the original data of the indicator were taken, and a regression model was built for the econometric test:

(1) $$\ln Y_{t}=A+\alpha \ln K_{t}+\beta \ln H_{t}+\gamma_{1}\ln L_{1t}+\gamma_{2}\ln L_{2t}$$

- Where Y_t_ denotes hospital output in year t, expressed in terms of hospital service quantities
- (Service quantities = total annual number of visits in hospitals + (annual average daily service charge / annual average outpatient fee) × total annual inpatient bed days)
- K_t_ denotes the hospital fixed capital investment in year t, expressed as the total value of fixed assets in that year
- H_t_ denotes the hospital bed input in year t, expressed as the number of hospital beds
- L_t_ denotes the hospital labor input in year t, expressed as the average number of visits per physician per year and the average number of inpatient bed days per physician per year.

In Formula (1), K and H represent the capital input in the production function, L represents the labor input, and Q represents residual input.

The Cobb–Douglas model, the cross-output distance function model, and the multi-output distance function model were estimated using STATA 16.0.

### Patient and public involvement

Although we support the importance of patient and public involvement, this study is an analysis of hospital operations, and involving them as members of this research study was impractical.

## Results

**Figure 1:**
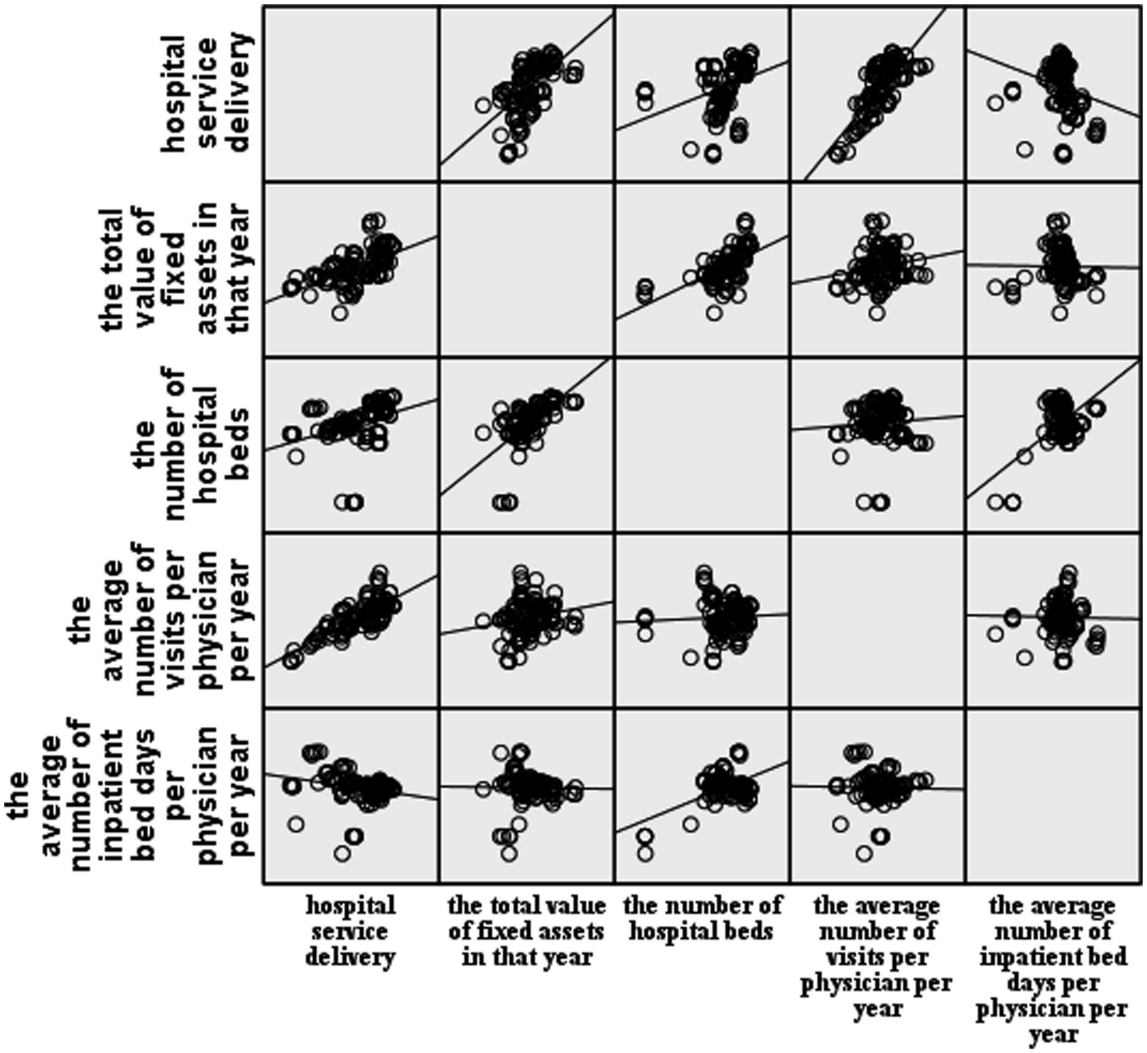
Scatterplot matrix.

The model was fitted using a stepwise regression method. According to the regression fitting results (Table 1), the complex correlation coefficient of model 4, *R* = 0.981, indicates that all four independent variables included are closely related to the service volume. In model 4, *R^2^* = 0.963 indicated that 96.3% of the degree of variation in service volume is explained by the four independent variables compared to the first three models that included only some variables. The increasing adjusted *R^2^* and decreasing residual standard deviation of the four models suggested an increasingly accurate model fit. Therefore, regression model 4 (*F* = 686.948, *p* < 0.01) was selected, where the regression coefficient of at least one independent variable was not zero, and the regression model was statistically significant. Figure 1 illustrates a mostly linear relationship between the four variables and the hospital service quantities. The residuals conform to a normal distribution, further suggesting a good model fit (Table 2).

**Table 1:** Regression model summary.

Model	*R*	*R^2^*	Adjusted *R*^2^	Std. Error of the estimate	Durbin-Watson (U)	*F*	*p*
1	0.806^a^	0.650	0.647	0.619		200.487	<0.001
2	0.916^b^	0.838	0.835	0.423		277.364	<0.001
3	0.940^c^	0.884	0.881	0.359		270.502	<0.001
4	0.981^d^	0.963	0.962	0.204	1.268	686.948	<0.001

^a^Predictors: the average number of visits per physician per year. ^b^Predictors: the average number of visits per physician per year, the total value of fixed assets in that year. ^c^Predictors: the average number of visits per physician per year, the total value of fixed assets in that year, the average number of inpatient bed days per physician per year. ^d^Predictors: the average number of visits per physician per year, the total value of fixed assets in that year, the average number of inpatient bed days per physician per year, the number of hospital beds.

**Table 2:** Residual normality test.

Std. error of the estimate	Kolmogorov–Smirnov Z	*p*
0.981	0.082	0.065

To further determine the model that fits each hospital, we substituted data from the 22 sample hospitals from 2016 to 2020 to include 4 variables: the average number of visits per physician per year, the total value of fixed assets in that year, the average number of inpatient bed days per physician per year, and the number of hospital beds. The coefficients for each item were determined in the regression model. Table 3 shows the model coefficients for the eight hospitals that are planning to conduct OHMC (A), the model coefficients for the six hospitals that are already conducting OHMC (B), and the model coefficients for the eight hospitals not planning to develop OHMC (C).

**Table 3:** 22 Public hospitals’ model construction.

Hospitals	*α* (K)	*β* (H)	*γ*_1_ (L_1_)	*γ*_2_ (L_2_)	Q	*α* + *β* + *γ*_1_ + *γ*_2_
**Planning to conduct OHMC**						
A1	0.589	3.019	1.072	−0.002	−18.122	4.678
A2	0.007	0.864	0.866	−0.111	6.480	1.626
A3	0.910	2.690	2.865	−2.252	−21.964	4.213
A4	−0.074	3.279	4.453	−3.427	−11.820	4.231
A5	0.01	−0.263	0.571	0.533	14.874	0.851
A6	0.694	——^a^	0.706	0.139	3.818	1.539
A7	−1.481	10.485	−0.644	1.489	−36.757	9.849
A8	3.519	0.857	1.007	0.008	−38.817	5.391
M1(*α* + *β* + *γ*_1_ + *γ*_2_)						4.047
**Already conducting OHMC**						
B1	0.543	1.636	2.406	−1.599	−10.298	2.986
B2	0.131	0.357	1.583	−0.521	6.573	1.550
B3	−3.641	−15.991	−1.050	3.235	162.006	−17.447
B4	0.746	5.202	2.673	−1.167	−39.545	7.454
B5	−0.337	0.702	0.675	0.300	12.500	1.340
B5	0.248	0.134	1.232	−0.604	7.620	0.974
M2(*α* + *β* + *γ*_1_ + *γ*_2_)						2.861
**Not planning to development OHMC**						
C1	0.944	2.638	1.898	−0.068	−20.558	5.412
C2	−0.027	−0.304	1.120	−0.192	13.411	0.597
C3	0.167	2.709	——^a^	0.626	1.645	3.502
C4	−0.781	−2.25	0.618	2.353	34.812	−0.06
C5	0.813	−4.15	4.242	2.019	21.666	2.924
C6	−0.007	0.853	−2.174	2.765	11.823	1.437
C7	2.325	−0.769	−0.058	0.818	−12.577	2.316
C8	−0.292	0.527	0.067	0.613	13.230	0.915
M3(*α* + *β* + *γ*_1_ + *γ*_2_)						2.130

^a^The indicator of the average number of inpatient bed days per physician per year did not change for five consecutive years, so it was excluded from the model fit and no specific coefficients could be calculated.

For example, the size measurement model for hospital A1 was lnY = 0.589lnK + 3.019lnH + 1.072lnL_1_ − 0.002lnL_2_ − 18.122, but the number of hospital beds in hospital A6 and the average number of inpatient bed days per physician per year in hospital C3 did not change for five consecutive years. They were excluded from the model fit, and the specific coefficients could not be calculated to obtain the definitive model.

According to the interpretation of the production function on the coefficient of output elasticity of labor and the coefficient of output elasticity of capital, let α + β + γ_1_ + γ_2_ = M. M > 1 indicates the incremental scale reward, where an input increase by a factor of 1 results in an output increase by a factor greater than 1. In other words, in this case, increasing the hospital size by a factor of 1 would result in higher output than building 2 hospitals of the same size. As displayed in Table 2, 72% of the 22 sample hospitals have the conditions to expand the scale into developing OHMC hospitals. Among them, the M-values of planning to operate OHMC hospitals ranged from 0.851 to 9.849, with the smallest M-value being hospital A5 and the largest M-value being hospital A7. The M-values of the OHMC hospitals in operation ranged from −17.447 to 7.454, with the smallest M-value being hospital B3 and the largest M-value being hospital B4. The M-values of the hospitals not planning to develop OHMC hospitals ranged from −0.06 to 5.412, with the smallest M-value being hospital C4 and the largest M-value being hospital C1. And there are M1 > M2 > M3, i.e., From the perspective of the economy of scale, the eight hospitals planning to operate OHMC hospitals are qualified to handle the conditions and potential of a multi-district organization. However, the hospitals already operating OHMC hospitals appear to have weakened incremental scale rewards (M2 < M1) because they are in the process of development but still have a higher M-value than single-campus hospitals (M2 > M3). This suggests that the new branch hospitals may have a short-term negative impact on the overall operational efficiency of the hospital, but will not cause long-term effects.

## Discussion

The M < 0 values of the B3 and C4 hospitals may be related to the special circumstances of the hospitals themselves. B3 is an obstetrics and gynecology hospital, and in order to develop women’s and children’s health in the surrounding areas of this city, B3 Hospital has established branch hospitals and escrowed several hospitals. Hospital trusteeship (33) is a loose management (34) model in which the government assigns a professional operation team to manage the day-to-day operation of the hospital. This model implies that host and managed hospitals have their own independent system and operate independently, unlike OHMC, which uses one management system. As a result, the hospital trustee model faces some difficulties in China. For example, the trend towards administrative leadership in public hospital management, the joint ownership of hospitals by multiple departments, and the lack of clear hierarchy and responsibility, especially for the management of the trustee hospital’s finances, which involves the real interests of both hospitals and patients, are all factors that may affect the efficiency of hospitals operating under the trustee model. C4 Hospital is a geriatric hospital that may not operate efficiently due to the particular patient population and its special geographical location, which may lead to poor operational efficiency.

In operating OHMC hospitals, hospital B4 shows the highest labor and capital elasticity coefficients (α + β + γ_1_ + γ_2_ = 7.454) and is also the hospital with branch hospitals where all branches are in normal operation. Therefore, it can be speculated that up to three hospital sites can be operated under the existing policy and overall city environment. Nevertheless, the feasibility of building more branches still needs further empirical studies.

According to a 1995 study by Hsing and Bond (35), a 272-bed, 945-employee hospital was found to be the most productive. In 1999, Hollingsworth et al. (36) analyzed the optimal hospital size in terms of input–output efficiency and found that a general hospital with 800 to 1,200 beds was the most appropriate size. In 2002, Polyzos (37) performed a correlation and regression analysis of hospital efficiency, revealing that district and general hospitals with 250–400 beds, and regional and teaching hospitals with 400 beds were highly efficient. In this study, we analyzed the number of beds in 22 sample hospitals, with an overall average number of beds of 1,038. The average number of beds in operating OHMC hospitals was 1,384. Combined with the overall planning of general hospitals in China and relevant literature studies, it can be considered that 1,000 to 1,400 beds is an appropriate size.

Regarding the suitability of hospitals to establish branch hospitals, the *Guiding Principles for Medical Institution Setting Planning (2021–2025)* (9) by the National Health Commission of the People’s Republic of China, gives a formula that can be quickly self-tested. The formula proposes a bed demand coefficient R (R = bed/actual number of open beds), in which R ≤ 1 indicates that hospitals are not suitable for building branch hospitals for the time being and should strengthen the internal construction of hospitals to further improve the efficiency of medical services; 1 < R < 1.3 indicates that the hospital should focus on improving construction, further improve medical services, and improve the efficiency of services, depending on the situation can be the talent pool; R ≥ 1.3 indicates that the hospital can develop branch hospitals to control the size of the existing single hospital depending on staff reserves.

However, due to a variety of realities, such as the wide geographical area and uneven population distribution in China, measuring the developmental stage of a hospital should also take into full consideration factors such as the service radius, service population, service demand, and operational efficiency of public hospitals. Therefore, the literature was reviewed, and government departments were consulted to select a model based on available indicators and construct a new formula. The latter makes up for the shortcomings of the above formula, covering incomplete content while not requiring a large number of indicators and simplifying data collection.

The adoption of the OHMC system generally faces challenges in the development process due to various factors such as institutional mechanisms, management philosophy and hospital culture. In addition to the current hospital operating condition, the management mode of the hospital also directly affects the time to operation after the establishment of the new branch. and whether it can achieve the “1 + 1 > 2” effect with the main hospital area in the future to promote each other. The Mayo (38) Clinic has adopted a centralized and decentralized management structure for the OHMC system, with a four-tier management structure, including a board of directors, a governance committee, a management team, and two operating teams. One of the two operating teams is responsible for the daily operation of the clinical, teaching and research, and commercial departments of the cross-district hospital, while the other is the operating team of each branch hospital. This approach not only allows each hospital and its affiliated medical service providers to adjust their operations according to their own market conditions but also ensures a unified overall culture, treatment and processes. Therefore, the same management philosophy is shared among hospitals in different regions. Taiwan Chang Gung Hospital (39) introduced the corporate management model of Taiwan Plastics Industry Co. to run the hospital as a business. In 1983, Chang Gung Hospital established the Medical Management Center, which was responsible for the construction of the entire hospital management system and operation management. The administration department reasonably allocated manpower and facilities according to the hospital’s operational needs after close accounting and effectively controlled the cost of hospital operations according to the patients attending the hospital, with cost control tasks specified to individuals. Chang Gung Hospital also adopted a patient-centered, horizontally integrated medical service, established a multi-disciplinary team of medical professionals, and each branch hospital integrated medical resources to set up medical centers for various diseases. These measures effectively compensated for the typically lower profits generated by general hospitals compared to specialty hospitals.

The OHMC system can adjust the spatial layout of hospitals, optimize the allocation of medical and health resources, and to a certain extent, promote the equalization of basic health services. Especially during the COVID-19 pandemic, as China applies a policy of “regular pandemic prevention and control,” hospitals need to provide both normal treatment and isolated treatment for confirmed cases of COVID-19. OHMC offers an obvious advantage in the context of an pandemic, as the government can quickly commandeer a branch hospital and convert it into a designated hospital for confirmed cases, thus not impairing access to normal medical care. The conversion of the main hospital to a designated pandemic hospital would inevitably affect the daily medical needs of the residents. However, an independent branch hospital could take up the task of pandemic prevention and control, and the daily business of the main hospital area can be carried out normally. The practice of pandemic prevention, control and treatment across China has highlighted the central role of quality medical resources, and establishing branch hospitals is essential to divide the functions of the main hospital. Furthermore, in case of other public health emergencies, such as natural disasters, branch hospitals can be used for emergency relief. In such situations, the advantages of the new branch hospital’s large space and complete support facilities can be brought into play, mobile cabin hospitals and medical teams can move in with ease, and even the facilities around the branch hospital can be put into use when necessary.

### Limitations

Due to the variability in the operating conditions of individual hospitals, as well as the differences in their geographical locations, disease spectrum and other factors, it is not possible to uniformly determine the coefficients (α, β, γ) in the above model and establish a general model applicable to all hospitals.

The sample hospitals include OHMC hospitals in operation, and the sub-hospitals were not analyzed in separate calculations. Since they were all viewed as a single hospital for measurement, it may have an impact on the results. Data from individual hospitals differed from usual due to China’s pandemic prevention policy and concerns about bias, so data from 2020 after the onset of COVID-19 were not included.

Since some of the hospitals that have established OHMC hospitals are still in the transition period of OHMC management, the results of OHMC hospital operations have not been analyzed. Analysis of the effects of OHMC hospital operations may need to be achieved through data analysis of hospital resource utilization efficiency, cost–benefit changes, etc. It is important to assess whether OHMC hospital operations can achieve the goals of branch hospital operations (governmental decision-making goals, medical service goals, and hospital scale expansion goals) for OHMC management. In the follow-up study, we hope to communicate and negotiate with hospitals to obtain relevant information and conduct research on the effects of OHMC hospital operations and goal achievement.

## Conclusion

The fundamental purpose of developing OHMC is to alleviate the need for patients to leave the district and relieve the pressure of tertiary hospitals in the city center. Therefore, several factors need to be examined in order to achieve a more effective organizational structure, including whether the medical tasks undertaken by the hospital are consistent with the local medical needs, whether the affordability of talents matches their development space, whether the number of hospital beds is coordinated with the local medical resource allocation, whether the comprehensive benefits of the hospital match the living standards of local residents, and whether the hospital scale expansion is compatible with its own environmental facilities. The expansion of hospital scale may promote group development, but it may also trigger rising hospital costs, increasing management and operation costs, inefficient allocation of medical resources and unbalanced development. Whether these important factors can be met at the same time and how to appropriately apply “scale expansion” is a question that hospital managers should consider in the management of OHMC.

## Data availability statement

The original contributions presented in the study are included in the article/supplementary material, further inquiries can be directed to the corresponding author.

## Author contributions

YZ and LJ contributed to the conception and design of the study. ZM organized the data collection. YZ performed the statistical analysis and wrote the content of the manuscript. YZ, ZY, and LJ contributed to the interpretation of the results and critical revision of the manuscript for important intellectual content and approved the final version of the manuscript. All authors contributed to the article and approved the submitted version.

## Funding

This research was supported by the Capital Health Management and Policy Research Base of China (2021JD04) and Beijing Hospital Management Center (2021SK000592).

## Conflict of interest

The authors declare that the research was conducted in the absence of any commercial or financial relationships that could be construed as a potential conflict of interest.

## Publisher’s note

All claims expressed in this article are solely those of the authors and do not necessarily represent those of their affiliated organizations, or those of the publisher, the editors and the reviewers. Any product that may be evaluated in this article, or claim that may be made by its manufacturer, is not guaranteed or endorsed by the publisher.
